# Early Clinical Outcomes of Short versus Long Proximal Femoral Nail Anti-rotation (PFNA) in the Treatment of Intertrochanteric Fractures

**DOI:** 10.5704/MOJ.2107.017

**Published:** 2021-07

**Authors:** JLM Loh, DME Huang, J Lei, W Yeo, MK Wong

**Affiliations:** 1 Department of Orthopaedic Surgery, Singapore General Hospital, Singapore; 2 Orthopaedic Diagnostic Centre, Singapore General Hospital, Singapore

**Keywords:** proximal femoral nail anti-rotation, intertrochanteric fractures, long PFNA, short PFNA, Asian population

## Abstract

**Introduction:**

Both short and long PFNA are employed to treat intertrochanteric fractures. Controversy exists in the choice between the two nails as each implant has specific characteristics and theoretical advantages. This retrospective study seeks to examine the operative complication rates and clinical outcomes of short versus long (Proximal Femoral Nail Antirotation) PFNA in the treatment of intertrochanteric fractures.

**Material and Methods:**

Between July 2011 and February 2015, 155 patients underwent PFNA insertion. The decision on whether to use a short or long PFNA nail, locked or unlocked, was determined by the attending operating surgeon. Visual Analogue Pain Score (VAS) Harris Hip Scores (HHS), Short-form 36 Health Questionnaire (SF-36) and Parker Mobility Scores (PMS) were collected at six weeks, six months and one year post-operatively.

**Results:**

A total of 137 (88.4%) patients were successfully followed-up. Forty-two (30.7%) patients received a short PFNA. The patients were similar in baseline characteristics of age, gender, and comorbidities. Operative time was significantly longer in the short PFNA group (62 ±17 mins) versus the long PFNA group (56±17). While the patients in both groups achieved improvement in all outcome measures, there was no significant difference between the groups in terms of HHS (61.0 ±16.0 vs 63.0 ±16.8, p=0.443), PMS (2.3±1.5 vs 2.7±2.1, p=0.545) and VAS (1.7±2.9 vs 1.8 ±2.2 p=0.454). There were 3 (7.1%) and 7 (7.4%) complications in the short versus long PFNA group, respectively.

**Conclusion:**

Both short and long PFNA had similar clinical outcomes and complication rates in the treatment of intertrochanteric fractures in an Asian population.

## Introduction

The incidence of hip fractures is increasing with the total number of hip fractures expected to surpass six million by the year 20501,2. Intertrochanteric fractures constitute one of the most common fractures of the hip, occurring mainly in elderly populations with osteoporosis^3^. Treatment of intertrochanteric fractures in elderly patients pose a huge challenge for orthopaedic surgeons due to the patients’ poor bone quality and significant comorbidities which increase the risks associated with surgery and anaesthesia^4^. Therefore, choosing the optimal fixation method and instrumentation is paramount to minimising complications and allowing early ambulation in this fragile patient population.

The Proximal Femoral Nail Anti-rotation (PFNA) designed by Arbeitsgemeinschaft für Osteosynthesefragen (AO Foundation) and distributed by Depuy Synthes © [Depuy Synthes, Warsaw, Indiana] is gaining popularity in the treatment of intertrochanteric fractures. This is especially true with the creation of new-generation implants which cater to patients of varying stature. Studies suggest that using the PFNA for intertrochanteric femur fractures may result in shorter operating times, decreased blood loss, greater fixation stability, shorter inpatient stay and potential protective effects with respect to possible future femur fractures^5,6^. Currently, both short and long versions of the device are employed to treat intertrochanteric fractures. However, controversy exists in the choice between the long and short PFNA as each implant has unique characteristics and theoretical advantages.

When the PFNA was first put into use, early studies showed that short nails were associated with a higher risk of secondary femur fracture^7^. This was theorised to be due to greater stress forces caused by the large and rigid distal end of the nail as opposed to a thinner tapered more flexible tip^8^. However, design modifications such as having a tapered end, smaller locking screws and using flexible implant materials such as titanium subsequently decreased the incidence of such fractures, and it is now thought that there is no difference in the propagation of such fractures regardless of the type of nail used^7^.

Patient anatomy also plays a role in the selection of PFNA used for intertrochanteric femur fractures. Currently, the long PFNA nails have straight and bowed versions while short PFNA nails only have straight versions. Chang *et al* believe that in patients with increased anterior bowing of the shaft i.e., Asian populations^9^, intertrochanteric femur fractures should be fixed with the long bowed PFNA for better fixation^10^. Other indications for the use of long nails include unstable fracture patterns such as fractures with subtrochanteric extension^11^ and fractures in patients who are at risk for distal secondary femur fractures^12^. Previous studies have shown similar rates of complications and reoperation with long nails, but increased blood loss and operative times^12-16^.

To our knowledge, only a few papers have assessed the clinical outcomes of short versus long PFNA in the treatment of intertrochanteric fractures in an Asian population. Thus, this study seeks to examine the clinical outcomes and operative complication rates of short versus long PFNA, in the treatment of intertrochanteric fractures in an urban Asian population at our Level One Trauma Centre.

## Materials and Methods

This is a retrospective study. Between July 2011 and February 2015, 155 patients underwent PFNA insertion for AO classification 31-A1 and A2 intertrochanteric fractures in our institution. Inclusion criteria were patients with low velocity trauma (same level fall), and surgical treatment for intertrochanteric hip fractures using PFNA nails. Patients with pathological fractures, high-energy traumatic fractures (such as road traffic accidents or a fall from a height), active malignancy, a significant history of thromboembolism or were transferred to other hospitals were excluded from the study.

Patients were given analgesia and optimised for surgery. Patient demographics, co-morbidities (hypertension, type 2 diabetes mellitus, end-stage renal failure), pre-injury ambulatory status, mechanism of injury and fracture location were recorded. Blood investigations detailing the patient’s pre-operative haemoglobin level, platelet count, serum urea and creatinine levels were also studied. Before surgery, patients underwent lateral femoral radiographs for estimation of canal size and degree of anterior bow of femur shaft, to aid in determination of the nail diameter and length. The decision on whether to use a short or long PFNA nail, locked or unlocked, was determined by the attending surgeon.

All operations were completed by an experienced orthopaedic surgeon with at least five years of experience treating orthopaedic trauma. Fracture reduction and insertion of the PFNA is carried out on a traction table, under image intensifier (I-I) guidance. The fracture is first reduced on the traction table and where possible, the affected limb is usually placed in 10° to 15° of adduction to facilitate nail insertion. Once adequate reduction is obtained, a 5cm skin incision is made about 5cm proximal to the tip of the greater trochanter. The fascia and gluteus medius are split in line with its fibres and a 3.2mm guide wire is inserted to locate a good entry point for the nail at the tip of the greater trochanter. The K-wire is advanced 10cm to 15cm into the femur and an I-I image is checked in AP and lateral to ensure good positioning before the opening ream is performed with protection of the overlying soft tissue. The nail size is estimated by using the template provided and the nail of chosen size is mounted on the insertion handle and introduced manually into the femur. For long nails, there is an additional step with insertion of a long ball-tipped wire down the femoral canal and over-reaming the femoral canal by 1.5mm before insertion of the long PFNA nail. The 130° aiming arm is attached to the insertion handle and through a 2cm lateral incision the guide wire for the PFNA blade is inserted under I-I guidance, aiming for an inferior/central position in the femoral head on the AP view and central position on the lateral view. The PFNA blade length is measured and attached to the inserter. The outer cortex is opened with a drill and the femoral neck and head are reamed with a cannulated reamer with a fixed fixation sleeve. The PFNA blade is inserted by light blows with the hammer. Once the PFNA blade position within the femoral head is satisfactory, the inserter is rotated clockwise to lock the PFNA blade to prevent rotation of the PFNA blade within the femoral head. Distal locking is then performed with the aid of the jig for short nails and distal locking may or may not be performed for the long nails under fluoroscopic guidance, depending on the surgeon’s preference.

The patients’ radiographs were reviewed again the second post-operative day. Physiotherapy was instituted on the second post-operative day and the patient was allowed to bear full weight after review of radiographs. All patients were also put on mechanical and chemical thromboprophylaxis which involved the use of intermittent pneumatic compression pump, thromboembolic deterrent open toe knee length compression stockings and oral anticoagulation therapy to prevent deep vein thrombosis (DVT). Mechanical prophylaxis was continued until patients were able to ambulate confidently with walking aids for two physiotherapy sessions on the same day.

Pre-operative parameters, as well as Visual Analogue Pain Score (VAS), Harris Hip Scores (HHS) and Parker Mobility Scores (PMS) at 6 weeks up to 1 year post-operatively were recorded. The outcome scores were obtained prospectively by independent assessors.

The data was prospectively collected at a centralised diagnostic centre and managed by an institutional joint registry with the requisite data protection and integrity protocols.

The study consisted of both descriptive and analytical components. Univariate analyses were performed to compare the post-operative outcome scores at six weeks and one-year post-operative between the patients who had short PFNA nail insertion and long PFNA nail insertion. Normality tests (Kolmogorov-Smirnov) were conducted on pre op and post op outcome scores which showed that these variables did not follow a normal distribution. For variables which are continuous and not of normal distribution such as the pre-op scores and post op scores, a Mann-Whitney U Test, a non-parametric analog of two sample t-test, was used to test whether two independent samples were drawn from the same population. This allowed us to obtain the asymptotic significance (2-tailed) p-value of the association of the variable to the outcome scores in our study. All analysis was performed using IBM Statistical Package for Social Sciences (SPSS) Version 22 [IBM, Armonk, NY, USA].

Ethical approval was obtained by the institution’s institutional review board ethics committee prior to commencement of the study. The reference number is 2016/2497.

## Results

Between July 2011 and February 2015, 155 patients underwent PFNA insertion. A total of 137 (88.4%) patients were successfully followed-up. Forty-two (30.7%) patients received a short PFNA with distal locking. The patients were similar in baseline characteristics of age (80.8±7.7 vs 80.1±8.6, p=0.630), gender, comorbidities, and clinical scores (Table I).

**Table I:** Baseline Characteristics

Sociodemographic	Short	Long	P value
Age			0.583
<60	1	2	
60-69	2	11	
70-79	11	35	
80-89	22	34	
>89	4	12	
Mean	80.8 ± 7.7	80.1 ± 8.6	0.630
Gender			
Male	8	36	
Female	34	59	
Race			
Chinese	35	78	
Malay	3	12	
Indian	3	4	
Others	1	1	
Comorbidities			
HTN	25	67	0.206
DM	18	46	0.547
IHD	10	31	0.299
HLD	19	45	0.818
Stroke	4	12	0.602
Arthritis	4	5	0.353
Asthma	1	7	0.251
Depression	2	1	0.171
Colitis	0	1	0.505
Psoriasis	0	0	
Parkinson’s Disease	2	3	0.644
Renal Impairment	2	9	0.349
Vascular disease Impaired cognition	0 5	0 14	0.658
Pre-Fall PMS	6.55	5.81	0.141
Pre-Fall VAS	0.10	0.23	0.412
Pre-Fall EQ Health	73.81	71.81	0.469
Pre Fall EQ Total	0.79	0.77	0.801
Pre-Fall SF-36 Total	75.48	74.71	0.818
Pre-Fall HHS Total	56.00	56.62	0.873

**Fig. 1:**
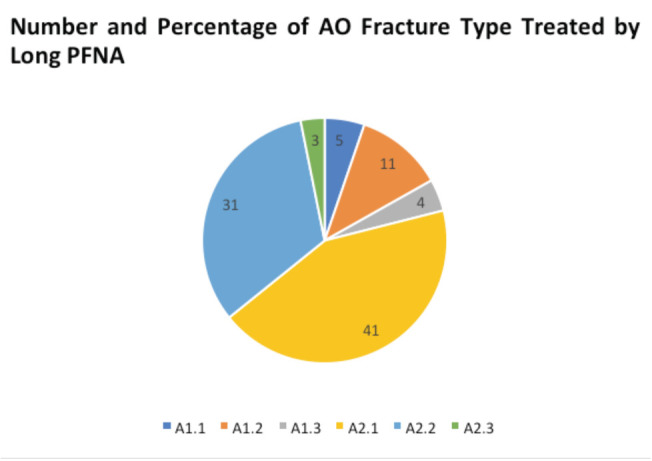
Number and Percentage of AO Fracture Type Treated by Long PFNA.

**Fig. 2:**
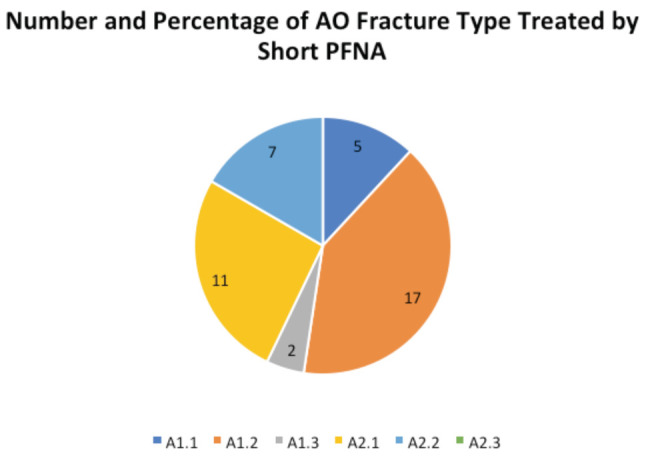
Number and Percentage of AO Fracture Type Treated by Short PFNA.

All short PFNAs were locked distally while 12 (12.6%) in the long PFNA group had distal locking performed. As this was for stable intertrochanteric fractures, the surgeons performing long PFNA nails without distal locking felt that there was no need for distal locking (reducing operative time) when there was good fit of the nail in the canal and the long nail spans the entire length of the femur. The operative time was significantly longer in the short PFNA group (62 ±17 mins) versus the long PFNA group (56±17mins) (p=0.023). Time taken for individual steps was not captured. The AO classification of the fractures encountered, and type of nails used to treat each type of fracture are shown in Table II and Fig. 1 and 2. The proportion of AO A2 fractures differed, exhibiting a predilection of long PFNAs for treating multi-fragmentary intertrochanteric fractures in our centre.

**Table II:** Functional Outcome Scores

	Short	Long	p-value
PMS Score			
Pre-Fall	6.55	5.81	0.141
6 weeks	1.46	1.65	0.518
12 weeks	2.28	2.69	0.295
6 months	2.92	3.29	0.487
12 months	3.50	4.23	0.244
VAS			
Pre-Fall	0.10	0.23	0.412
6 weeks	1.94	2.32	0.464
12 weeks	1.86	1.79	0.880
6 months	0.85	1.63	0.137
12 months	1.15	0.63	0.214
EQ Health			
Pre-Fall	73.81	71.81	0.469
6 weeks	66.00	70.63	0.172
12 weeks	68.06	71.62	0.269
6 months	70.77	69.74	0.786
12 months	68.08	70.69	0.516
EQ Total			
Pre-Fall	0.79	0.77	0.801
6 weeks	0.15	0.25	0.251
12 weeks	0.25	0.45	0.071
6 months	0.42	0.46	0.743
12 months	0.46	0.53	0.580
SF-36 total			
Pre-Fall	75.48	74.71	0.818
6 weeks	50.44	49.31	0.722
12 weeks	54.33	54.88	0.966
6 months	58.96	59.54	0.884
12 months	60.92	65.60	0.337
HHS Total			
6 weeks	56.00	56.62	0.873
12 weeks	60.96	63.00	0.592
6 months	64.05	67.00	0.460
12 months	69.12	72.81	0.321

The mean time to ambulation (days) was 15.6 ±18.6 in the short PFNA group vs 18.8±37.6 in the long PFNA group (p=0.935). There were 3 (7.1%) complications and 7 (7.4%) complications in the short versus long PFNA group, respectively. Table III illustrates the different complications in each PFNA group.

**Table III:** Complications of Fixation with Short and Long PFNA

	non-union	cutout	non-union and cutout	distal fracture	distal screw break	periscrew fracture	superficial infection	deep infection
short	0	1	1	0	0	0	1	0
long	1	2	1	2	1	1	0	0

**Fig. 3:**
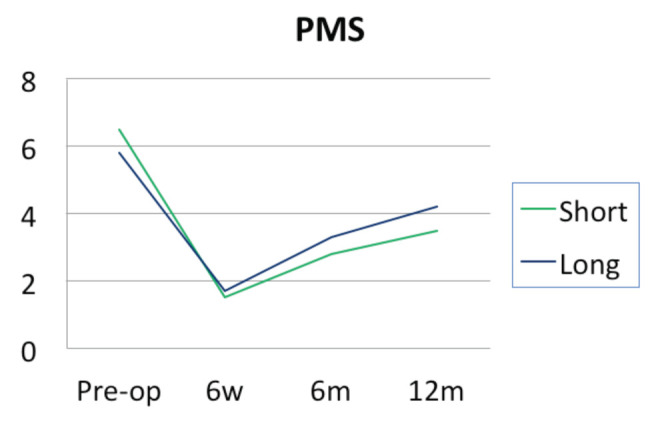
Pattern of the PMS Score in patients who had short nail fixation and patients who had long nail fixation.

While the patients in both groups achieved improvement in all outcome measures at six weeks and sustained to one year, there was no significant difference between the groups (short versus long, respectively) in terms of HHS (61.0 ±16.0 vs 63.0 ±16.8, p=0.443), PMS (2.3±1.5 vs 2.7±2.1, p=0.545) and VAS (1.7±2.9 vs 1.8 ±2.2 p=0.454). This is demonstrated in Table II. Fig. 3 illustrates the similar pattern of the PMS Score in patients who had short nail fixation and patients who had long nail fixation.

## Discussion

Our study demonstrated a longer operative duration in the short PFNA group and similar post-operative functional outcome scores between the short and long PFNA groups. However, we did not capture the time taken for individual steps.

One of the main reasons for the longer operative duration in the short PFNA group is the additional step of distal locking. This is consistent with previous studies which show that long PFNA nail insertion have been shown to have a shorter operation time due to the decreased need for distal locking^17^. Distal locking of nails increased operative time as there is an additional component to be added, with supplementary exposure required for insertion^18^. Previous studies have shown that the exact indications for locking a long IMN are not fully understood. As this was for stable intertrochanteric fractures, our surgeons performing long PFNA nails, like Ozkan *et al* believed that by avoiding the use of distal locking screws, they had the added advantage of decreased operation and fluoroscopy exposure time, increased patient mobility due to less tissue dissection and a low probability of iliotibial tract irritation due to the omission of a distal screw^19^. Additionally, our surgeons felt that there was no need for distal locking when there was already a good fit in the canal from the long nail, which also spans the entire length of the femur. This confers an additional benefit in elderly patients prone to falls as this would prevent an ipsilateral fracture in the same femur due to the strength conferred by the presence of a long intramedullary nail. Even if the ipsilateral femur does fracture from another fall, the solution would be to simply add on distal locking screws at that next fracture episode, as opposed to a revision from a short IMN to a long IMN or plating of a peri-implant short IMN fracture. In another study where distal locking was used for all nails, the long nails had a significantly longer operative time likely due to the longer preparation time and reaming time required for long nails^20^.

Significantly, patients in both groups achieved improvement in all outcomes measured up to 1 year and there was no significant difference between the groups. This could be because, unlike previous studies, our study shows no difference in the incidence of complications or length of stay between the two groups. Previous studies show patients with short nails having a higher incidence of secondary femur fractures when compared to long nails^7^. From the literature, the rate of proximal implant failure should theoretically be the same because the femoral head lag screw construct is identical, whether using a short or long intramedullary device^21^. Theoretically, a short nail causes a stress-riser just distal to the end of the nail while a long nail provides a protective effect to the entire femur which could affect the result in patients with osteoporotic bone^22^. This correlated with what our surgeons believe in, especially since our Asian population has a significantly higher incidence of anterior femoral bowing, resulting in the distal tip of short nails abutting the anterior femoral cortex. Sears *et al* state that as the long nail gains fixation at the isthmus and proximal locking bolt (which is placed near the lesser trochanter), relative stability is improved compared with the short nail, which is fixed at two closely spaced proximal locking holes^23^. However, Norris *et al*^7^ performed a systematic review of 13,568 patients in 89 studies between 1980 and 2010 and found that the risk of secondary femur fracture in short nails was not statistically significant. This is again reinforced by a recent study which shows that when comparing rates of all catastrophic failures between short and long nails, Vaughn et al found no statistical difference^24^. Our study also separates itself from previous studies which show differences in length of stay. This may be due to differences in the health status of patients in the different studies. For instance, in one of the previous studies, a higher percentage of diabetic patients were in the cohort receiving long nails^25^. Diabetic hip fracture patients have previously been reported to have increased lengths of hospitalisation compared to nondiabetic patients^26^. In a review of patients who sustained hip fractures of all patterns and who were treated with a variety of implants (PFNAs, dynamic hip screws, hemiarthroplasty, or cannulated screws), the length of stay in the hospital was associated with a higher ASA grade^27^. In this study, patients who received short and long nails had similar comorbidities which were shown not to affect the choice of nails.

Another important point to note is that while the clinical outcome scores were similar, the proportion of AO A2 fractures differed, suggesting the predilection of long PFNAs for treating multi-fragmentary intertrochanteric fractures. The predilection for choosing longer PFNA nails in our centre for treating more unstable fracture patterns could be due to the recognition of the more favourable biomechanical profile of longer PFNA nails^28^. A biomechanical study comparing the use of short and long PFNA nails showed that the femur medial stress peaks of the long PFNA were reduced significantly in comparison with those of the short PFNA^29^. Moreover, as previously mentioned, Asian populations have increased anterior bowing of the shaft and thus there is a preference for intertrochanteric femur fractures in Asian populations to be fixed with the long bowed PFNA^9^. This underlying paradigm possibly skewed our surgeons’ preferences.

This study has several strengths and limitations. The study included multiple types of fracture patterns with a diverse study population, used independent outcome assessors and is the first of its kind to assess post-operative functional outcomes between short and long PFNA. However, the fact that it is a single institutional series with a lack of randomisation may preclude extrapolation to other populations. Additional studies should be performed to determine the functional outcomes in other local health care systems, as well as the long-term functional outcomes and quality of life associated with the use of short and long nails. In conclusion, short and long PFNAs had similar early clinical outcomes and complication rates in the treatment of intertrochanteric fractures in this study. A future randomised study stratified by stability of fracture would be beneficial in investigating the superiority of long PFNA in unstable fracture patterns.

## Conclusion

Both short and long PFNA insertion had similar early clinical outcomes and complication rates in the treatment of intertrochanteric fractures.
